# Ligand binding and conformational dynamics of the *E. coli* nicotinamide nucleotide transhydrogenase revealed by hydrogen/deuterium exchange mass spectrometry

**DOI:** 10.1016/j.csbj.2022.09.036

**Published:** 2022-09-26

**Authors:** Jonathan Zöller, Sangjin Hong, Martin L. Eisinger, Malcolm Anderson, Melanie Radloff, Kristina Desch, Robert Gennis, Julian D. Langer

**Affiliations:** a Proteomics, Max Planck Institute of Biophysics, Frankfurt am Main, Germany; b Department of Biochemistry, University of Illinois, Urbana, USA; c Present address: AIMS, AbbVie Deutschland, GmbH & Co. KG., Ludwigshafen, Germany; d Waters Corporation, Stamford Avenue, Altrincham Road, Wilmslow SK9 4AX, United Kingdom; e Molecular Membrane Biology, Max Planck Institute of Biophysics, Frankfurt am Main, Germany; f Proteomics, Max Planck Institute for Brain Research, Frankfurt am Main, Germany

**Keywords:** Membrane protein, Channel opening, Small ligand binding, HDX-MS, Cyclic IMS-MS

## Abstract

Nicotinamide nucleotide transhydrogenases are integral membrane proteins that utilizes the proton motive force to reduce NADP^+^ to NADPH while converting NADH to NAD^+^. Atomic structures of various transhydrogenases in different ligand-bound states have become available, and it is clear that the molecular mechanism involves major conformational changes. Here we utilized hydrogen/deuterium exchange mass spectrometry (HDX-MS) to map ligand binding sites and analyzed the structural dynamics of *E. coli* transhydrogenase. We found different allosteric effects on the protein depending on the bound ligand (NAD^+^, NADH, NADP^+^, NADPH). The binding of either NADP^+^ or NADPH to domain III had pronounced effects on the transmembrane helices comprising the proton-conducting channel in domain II. We also made use of cyclic ion mobility separation mass spectrometry (cyclic IMS-MS) to maximize coverage and sensitivity in the transmembrane domain, showing for the first time that this technique can be used for HDX-MS studies. Using cyclic IMS-MS, we increased sequence coverage from 68 % to 73 % in the transmembrane segments. Taken together, our results provide important new insights into the transhydrogenase reaction cycle and demonstrate the benefit of this new technique for HDX-MS to study ligand binding and conformational dynamics in membrane proteins.

## 1 Introduction

The energy-coupled transhydrogenase is found in a variety of prokaryotes and in the inner mitochondrial membrane of most eukaryotes, except plants and yeasts [1], [2]. It plays an important role in cellular processes such as reducing oxidative stress, anabolic reactions and apoptosis [1], [2], [3]. As a membrane-embedded protein complex, the transhydrogenase utilizes the proton motive force (PMF) present across the membrane to generate NADPH while transporting one proton from the positive side of the membrane (mitochondrial intermembrane space or bacterial periplasm) to the negative side (mitochondrial matrix or bacterial cytoplasm). The enzyme appears to function as a proton transporter in which a protonatable histidine residue located near the middle of the membrane is alternately exposed to the positive and negative sides of the membrane, allowing protonation from one side and deprotonation to the other side.

$$\text{NADH}+\text{NAD}{\text{P}}^{+}+H_{\text{out}}^{+}⇄\text{NA}{\text{D}}^{+}+\text{NADPH}+H_{\text{in}}^{+}$$

**Fig. 1:**
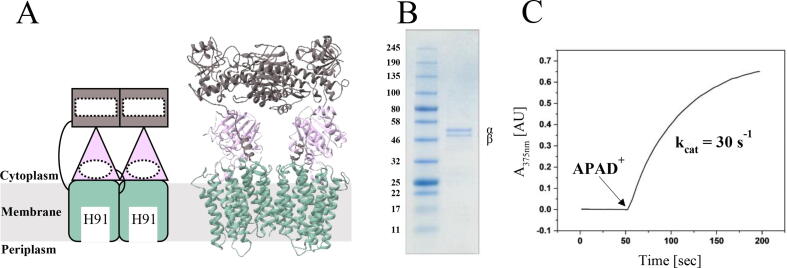
Activity assay of purified *E. coli* transhydrogenase*.* (A) Schematic and homology model of the *E. coli* transhydrogenase showing the two protomers of the homodimer. (B) The SDS-PAGE was conducted on 4–20 % precast PAGE gel with Coomassie brilliant blue staining. The upper and lower bands on the gel represent the PntA (α) and PntB (β) subunits, respectively. This gel shows excellent sample purity. (C) The reverse transhydrogenase activity was measured spectrophotometrically by following the reduction of APAD^+^ by NADPH at 375 nm at 37 °C using an Agilent 8453 UV–visible spectrophotometer and an absorbance coefficient of 6.07 mM^-1^cm^−1^. The activity assay was performed with DDM as detergent and showed that the transhydrogenase is active in solution. (For interpretation of the references to color in this figure legend, the reader is referred to the web version of this article.)

The membrane-bound transhydrogenase from different species has a conserved structure consisting of two identical protomers in which each protomer has three domains [1], [3], schematically illustrated in Fig. 1A. Domain I contains the NAD(H) binding site whereas NADP(H) binds in domain III [4], [5], [6], [7]. These two domains can form an interface where direct hydride transfer from NADH to NADP^+^ (forward reaction) occurs [1], [6], [7], [8]. Domain II is the transmembrane segment which contains the putative proton channel with a single protonatable histidine residue located near the middle of the membrane [1], [6]. Each protomer has the three domains stacked with domain III (NADP(H)-binding) in between domain II (proton channel) and domain I (NAD(H)-binding). In different species, each protomer consists of either one, two or three different subunits which encode the three conserved domains [1].

A low-resolution X-ray structure of the transhydrogenase from the hyperthermophilic bacterium *Thermus thermophilus* shows that domain III can exist in at least two very different conformations in which bound NADP(H) is either facing domain I, poised for direct hydride transfer to NAD(H), or facing domain II and the proton channel [9]. The only atomic-resolution structures of the entire holo-transhydrogenase have been obtained using cryo-electron microscopy of the transhydrogenase from ovine mitochondria [6]. The structures of the mitochondrial transhydrogenase show substantial conformational changes allosterically induced by binding of the NADP^+^ or NADPH to domain III. In the current work, the ligand-induced conformational changes of the transhydrogenase from *E. coli* are explored using hydrogen/deuterium exchange mass spectrometry (HDX-MS). The results show global structural changes to the binding of NADP^+^ and NADPH to the bacterial enzyme.

Considerable biochemical data are available for the transhydrogenase from *E. coli* along with X-ray structures of hydrophilic domains (I and III), but no structure of the entire enzyme is available. Sequence analysis [1], [10], X-ray and NMR structures of the hydrophilic domains I and III [4], [7], [11], [12], [13], [14], and homology modeling based on the structure of the mitochondrial enzyme [6] have been utilized to build a model of the *E. coli* transhydrogenase. In contrast to other bacterial enzymes, dI is connected with a linker to dII, which could make it possible for the transhydrogenase to exist in a double face down conformation. This double face down conformation is too found in the mammalian transhydrogenase, supporting a possible existence of a double-face down conformation in *E. coli* [6]. Each protomer of the *E. coli* enzyme consists of two subunits. The α subunit contains domain I with the NAD(H) binding site as well as the first four transmembrane helices (TM1–TM4) of the membrane domain II. The β subunit contains the additional 9 transmembrane helices of domain II (TM6–TM14; total 13 transmembrane helices) plus domain III with the NADP(H) binding site. TM3, TM9 and TM13 form the core of the putative proton channel surrounded by TM4, TM10 and TM14 [1], [6], [15], [16]. The only protonatable amino acid within the proton channel is His91 in TM9 and this residue is critical to transfer protons across the membrane [1], [6]. The NADP(H) binding site, located in domain III, is arranged in a Rossmann fold [1], [11], [17]. Several segments such as loop E and loop D are involved in NADP(H) coordination and define the substrate specificity for NADP(H) over NAD(H) [1], [4], [8], [18].

Over the last years, HDX-MS became a widely used method to analyze ligand binding and structural dynamics in proteins [19], [20]. The technique utilizes the exchange of backbone amide protons with surrounding solvent, which depends on temperature, pH, hydrogen bonding and solvent accessibility [21], [22]. Monitoring deuterium incorporation after incubation of the protein in D_2_O and contrasting changes in deuterium uptake in the presence or absence of a ligand provide information on binding sites and conformational dynamics. HDX-MS has become a routine method for soluble proteins [23], however membrane proteins remain a challenging target for structural studies because of their biophysical properties [22], [23], [24]. Since transmembrane segments are typically buried within a membrane or detergent micelle, they display lower deuterium uptake, and it is difficult to detect the uptake differences due to the more subtle differential available. The situation is exacerbated by the presence of higher levels of interfering ions, resulting from either lipids or detergents [22], [24]. Recent developments in biochemical preparation (reviewed in [25], [26]) and digestion [27], [28], [29] as well as liquid chromatography and MS data acquisition and analysis [30], [31] have significantly improved data quality.

Here we made use of a novel cyclic ion mobility separation (IMS) system to further improve resolution, coverage, and sensitivity for transmembrane segments in our HDX-MS data. Specifically, the higher sensitivity and ion mobility resolution enabled us to track smaller differences in deuterium uptake. We provide the first comprehensive map of the ligand-induced gating of the transmembrane proton channel and directly visualize ligand binding sites and the proton path through the membrane.

## 2 Methods

### 2.1 Purification of *E. coli* transhydrogenase

*E. coli* membranes containing wild-type transhydrogenase were thawed and dissolved in 2% DDM containing buffer to a final protein concentration of 4 mg/mL. Next, the protein was purified with a gravity flow Ni-NTA agarose column and pooled fractions were subjected to size exclusion chromatography (SEC). SEC was performed using an Äkta pure chromatography system with a HiLoad 16/600 Superdex 200 pg column. Protein-containing fractions were pooled, aliquoted in 100 µL fractions and stored at −80 °C for further experiments.

### 2.2 Activity assay in detergent

The reverse transhydrogenase activity was measured spectrophotometrically by following the reduction of acetylpyridine adenine dinucleotide (APAD^+^) by NADPH at 375 nm at 37 °C using an Agilent 8453 UV–visible spectrophotometer and an absorbance coefficient of 6.07 mM^-1^cm^−1^. The reaction mixture included 25 mM HEPES (pH 7.5), 50 mM KCl, 125 μg/ml asolectin, 0.1% DDM and 100 μM NADPH, and the reaction was started by adding 100 μM APAD^+^.

### 2.3 HDX sample preparation and mass spectrometry

Protein samples were freshly thawed and the buffer was exchanged to equilibration buffer (E-buffer; 20 mM HEPES, 150 mM NaCl, 0.02% DDM at pH 7.8) containing 1 mM of the respective ligand (binding) or without any ligand (control). The final sample concentration for the transhydrogenase was adjusted to 1.5 mg/mL.

HDX mass spectrometry experiments were carried out on a fully automated HDX-2 system (supplied by Waters, Milford USA) previously described in [22]. In brief, the sample was diluted 15-fold with the corresponding, fully deuterated, labeling buffer (L-buffer; 20 mM HEPES, 150 mM NaCl, 0.02% DDM at pH 7.4/pD 7.8) and incubated for specific times (0, 30, 180, 900 and 2750 s). The H/D exchange was quenched by diluting the sample in a 1 to 1 ratio in an acidic, ice cold (0 °C), phosphate buffer (150 mM KP_i_, 0.02% DDM at pH 2.2). Then, 24 pmol of protein was injected into the chromatographic system. After online peptic digestion, trapping and C18 separation, the peptides were measured on a Synapt G2-Si mass spectrometer (supplied by Waters, Milford USA) in HDMS^E^ mode (50–2000 *m*/*z*), including ion mobility for gas phase separation. Samples were measured in technical quadruplicates in randomized order.

To evaluate the system and measure protein-dependent deuterium back-exchange, we produced a fully deuterated reference sample and subjected it to the normal HDX-MS workflow.

### 2.4 HDX cyclic ion mobility mass spectrometry

*E. coli* transhydrogenase protein samples were prepared at a concentration of 4.0 mg/mL in phosphate storage buffer and transported on dry ice from MPI laboratories to Waters Corp. (Wilmslow, UK). Prior to use, the samples were diluted to 1.0 mg/mL using E-buffer. Aliquots containing 16 pmol of protein were injected onto the pepsin column.

Details were the same as previously described. Peptides eluting from the analytical reversed phase column were infused into the electrospray source of a SELECT SERIES^TM^ Cyclic^TM^ IMS mass spectrometer (Waters). Data were acquired in HDMS^E^ mode (50–2000 *m*/*z*), with the cyclic ion mobility device operating a single pass separation routine.

### 2.5 Data evaluation and statistics

Peptides were identified from non-deuterated measurements using ProteinLynx Global Server 3.0.3. (PLGS, Waters) for each condition (control and binding). Mass spectra evaluation was performed using the software DynamX 3.0 (Waters). For statistically relevant differences in deuterium uptake all peptides and their respective spectra were subjected to a two-stage *t*-test previously described by Houde *et al.* [32]. Statistics were performed with an in house written R-script previously described in [33]. All peptides that passed these two stages were used to create differential deuterium uptake maps (colour code according to reference [22] from red, decreased, over white, no change, to blue increased deuterium uptake), which were displayed on the most recent homology models of *E. coli* transhydrogenase built using UCSF Chimera [34] and based on the double face-down (attached) conformation of the ovine mitochondrial transhydrogenase.

Further and detailed information’s about experimental conditions and data evaluation are found in the supplement methods. The mass spectrometry proteomics data have been deposited to the ProteomeXchange Consortium (https://proteomecentral.proteomexchange.org) via the PRIDE partner repository [41].

## 3 Results

### 3.1 Enzymatic activity and sequence coverage of purified *E. coli* transhydrogenase

SDS-PAGE analysis of the purified enzyme showed high purity on the gel, displaying two distinct bands of PntA (α) and PntB (β) subunits corresponding to their expected molecular weight of 57.1 kDa and 48.7 kDa, respectively (Fig. 1B). The reverse transhydrogenase activity of the protein in DDM was measured using the reduction of APAD^+^ by NADPH. The result of this assay showed that the purified transhydrogenase was functional with a k_cat_ of about 30 s^−1^ (Fig. 1C). After optimizing conditions for proteolytic digestion in the HDX-MS experiments, we achieved more than 80% sequence coverage for PntA and over 75% for PntB (Fig. S1-5). Sequence coverage and redundancy were not sufficient enough to achieve single amino acid resolution, hence we analyzed deuterium uptake differences with peptide resolution.

### 3.2 NADPH binding

**Fig. 2:**
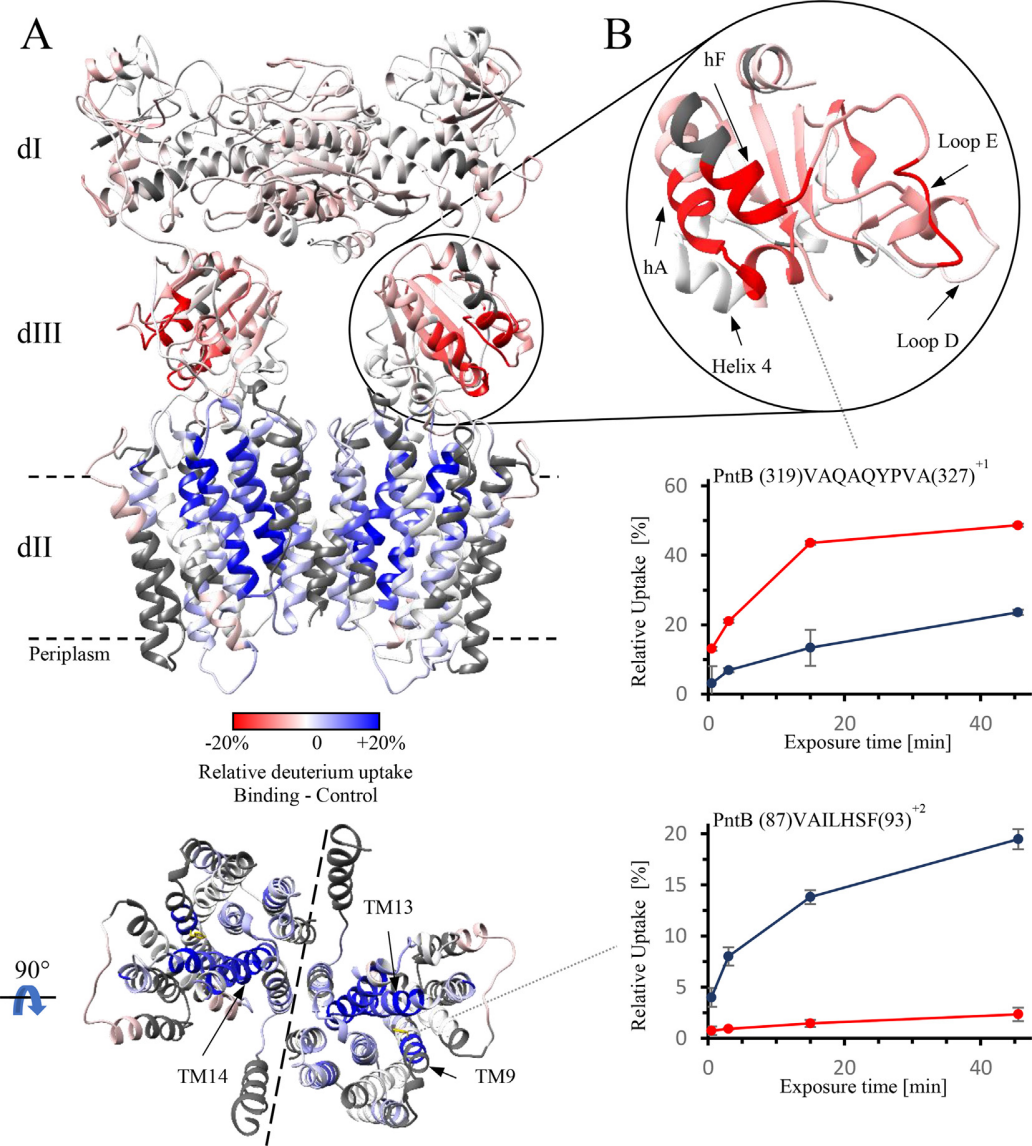
Differential deuterium uptake of *E. coli* transhydrogenase upon NADPH binding. (A) Deuterium incorporation of transhydrogenase was measured after incubation for 45.5 min in the presence (binding) or absence (control) of 1 mM NADPH. The differential deuterium uptake (binding - control) is displayed on homologue model structures of *E. coli* transhydrogenase in a “double face down” conformation. Differences are highlighted by a color gradient, ranging from red where uptake decreased over white, if no change occurred, to blue, indicating an increased uptake. *E. coli* transhydrogenase is shown parallel to the membrane as ribbon representation (top). A different orientation of domain II, from the cytoplasmic side, is shown in the bottom. The dotted line indicates the interface of the two protomers. Helices involved in formation of the putative proton channel are indicated and the important histidine 91 in TM9 is displayed in yellow. (B) Effects of NADPH binding in domain III are shown in more detail, where important segments involved in NADPH binding, are highlighted by arrows (upper right). This domain shows marked decreased deuterium uptake in key segments like loop E and helices hA and hF. Deuterium uptake plots of example peptides displaying different kinetics of deuterium incorporation are shown in the bottom. Error bars show the standard deviation of the relative deuterium uptake for each time point. (For interpretation of the references to color in this figure legend, the reader is referred to the web version of this article.)

Relative deuterium uptake was analyzed for all time points in the presence of 1 mM NADPH and compared to the apo-transhydrogenase reference sample. Fig. 2A shows the relative changes of deuterium uptake after 45.5 min mapped onto a homology model of the *E. coli* transhydrogenase. NADPH binding led to pronounced differential deuterium uptake in the protein. We observed a strong increase in deuterium uptake in key transmembrane helices in domain II: TM9, TM13 and TM14, as shown in Fig. 2A (bottom). TM9 contains residue His91, essential for proton transport. In contrast, domain III, which contains the NADP(H) binding site, exhibited decreased deuterium uptake in peptides involved in NADPH binding [4], [6]. These peptides are located in Loop D and Loop E, including the KRS motif (PntB residues 424–426), and its neighboring helices hA and hF (PntB residues 316–335 and 450–456, Fig. 2B) [1], [13]. One helix in the Rossmanfold in domain III (PntB 354–362) displayed no significant changes whereas the adjacent helix displayed a slightly decreased deuterium uptake. Segments around residue Y325 (PntB), which is located close to the NADPH binding pocket and can form a hydrogen bond with R350 (PntB), showed a strong decrease in deuterium uptake [6]. In domain I, we observed only a slight decrease in deuterium uptake around the Rossmann fold containing the NAD(H) binding site.

### 3.3 Tracking subtle changes in deuterium uptake with cyclic ion mobility

**Fig. 3:**
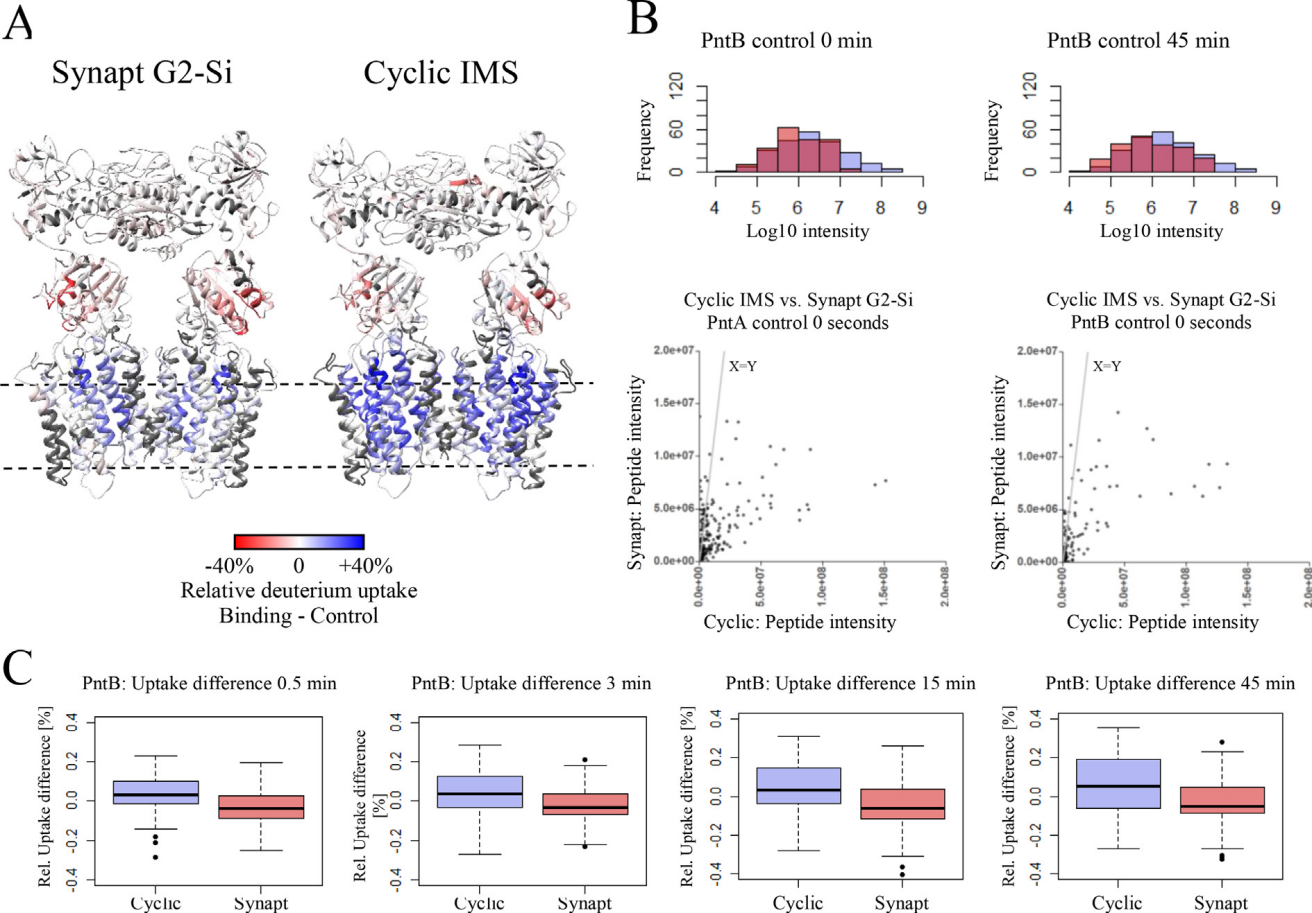
Comparison of NADPH binding to *E. coli* transhydrogenase acquired on Synapt G2-Si and SELECT SERIES Cyclic IMS mass spectrometer. (A) Deuterium uptake rate differences of NADPH binding to *E. coli* transhydrogenase measured on Synapt G2-Si (left) and SELECT SERIES cyclic IMS-MS (right) mass spectrometers and mapped onto the homology model structure. Transhydrogenase is shown in stereo parallel to the membrane as ribbon representation. (B) Intensity comparison of Synapt vs cyclic IMS-MS of selected time points. Histograms (top) show the log_10_-scaled peptide intensity as acquired for two selected time points (0 min or 45 min) on the Synapt (red) or the cyclic IMS-MS (blue). Scatter plots (bottom) compare peptide raw-intensities as measured on the two different instruments. Plots for all time points and subunits are shown in supplementary Figs. S6–S7. (C) Box plots indicate the distribution of peptide-wise deuterium uptake differences for both mass spectrometers for all four labeling time points upon NADPH binding in subunit PntB (Box plots of subunit PntA are shown in Fig. S13). (For interpretation of the references to color in this figure legend, the reader is referred to the web version of this article.)

We then set out to maximize deuterium uptake information for the membrane-embedded segments using a new cyclic ion mobility mass spectrometer. Measurements with this instrument resulted in similar peptide numbers and redundancy, despite less protein injected. In addition, the sequence coverage of transmembrane segments increased from 68% to 73% and including now important segments. Deuterium uptake differences in the cyclic IMS data set were significantly higher for the whole protein complex compared to the Synapt G2-Si data set (Fig. 3C and Fig. S18). Peptide raw intensities in the mass spectra were one order of magnitude higher in all time points compared to Synapt G2-Si data (Fig. 3B, Figs. S16 and S17) resulting in better S/N ratio.

Domain I displayed a slight decrease in deuterium uptake, mainly located in the area of the NAD(H) binding pocket. Domain III, harboring the NADP(H) binding site, showed decreased deuterium uptake, especially in the loop D and loop E, both of which are involved in ligand binding. Also, helices hF and hA displayed strong decreased uptake [13].

The strongest effects were observed in domain II which displayed an increase in deuterium incorporation in TM9, TM10, TM13 and TM14. These effects are much more pronounced and are now present over the entire helices, in contrast to the initial dataset acquired on the Synapt G2-Si in which only individual segments of these helices showed a change (Fig. 3A). In addition, we now observed an increase in helix TM4, not detected in the previous NADPH experiment with the Synapt G2-Si.

### 3.4 NADP^+^ and NAD^+^ binding

**Fig. 4:**
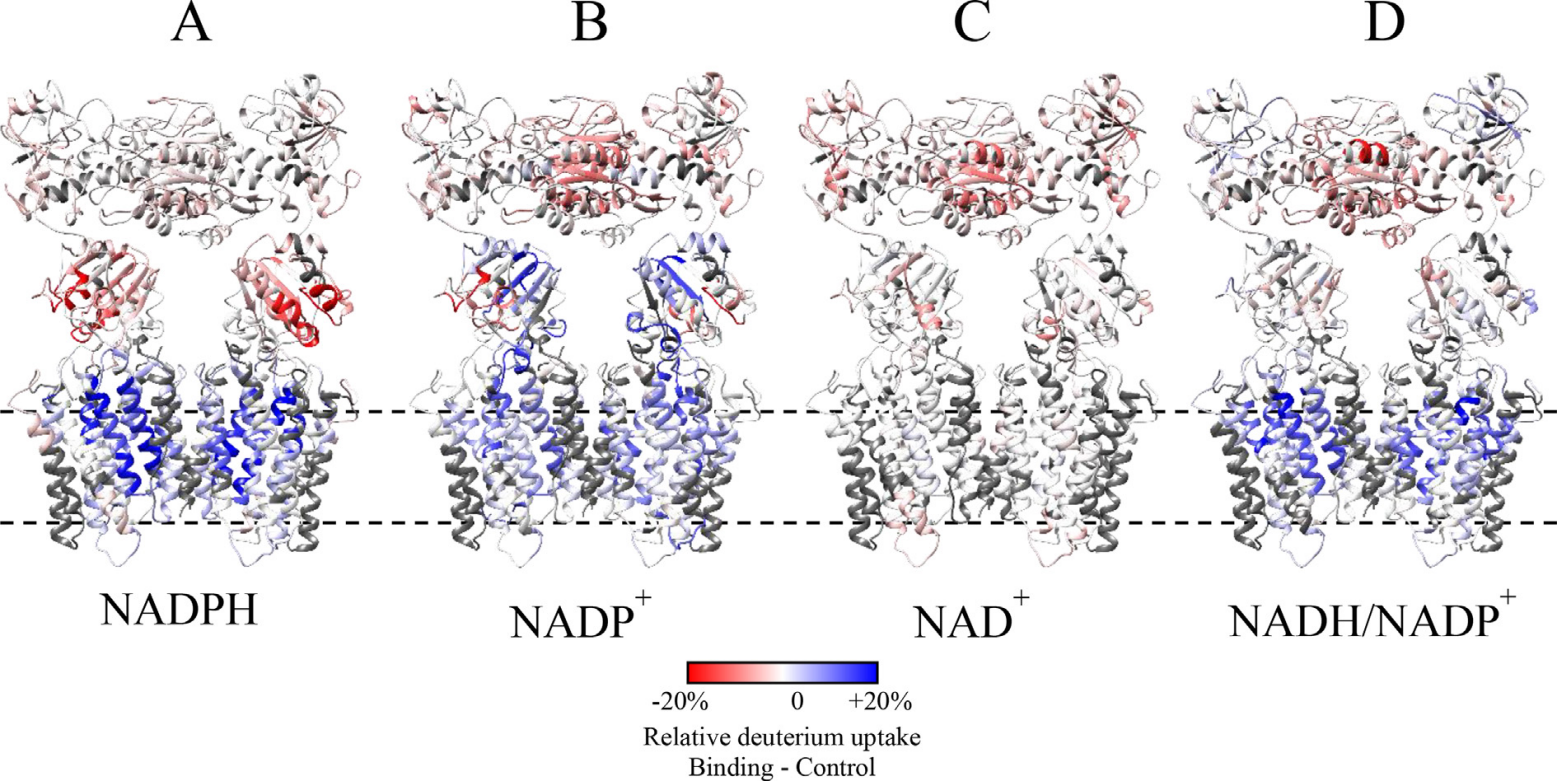
Overview of ligand binding experiments of *E. coli* transhydrogenase. Overview of differential deuterium uptake of *E. coli* transhydrogenase binding NADPH (A), NADP^+^ (B), NAD^+^ (C) and NADP^+^/NADH (D) showing different effects on the putative proton channel measured with a Synapt G2-Si mass spectrometer. *E. coli* transhydrogenase is shown as ribbon representation in a “double face-down” conformation [33]. Color gradient as in Fig. 2.

In the presence of 1 mM NADP^+^, the *E. coli* transhydrogenase is proposed to adapt a different conformation compared to the apo-transhydrogenase. The map of those changes is shown in Fig. 4B, compared to the results of all ligand binding experiments (NADPH, NAD^+^ and NADH/NADP^+^). NADP^+^ binding to domain III results in a mixed pattern of deuterium uptake rate in domain III. Key ligand binding segments have a reduced deuterium uptake rate (red) while several segments around helix 4 displayed an increased deuterium uptake (blue). A significant decrease in the rate of deuterium uptake was also observed in domain I near the interface between the two protomers of domain I. Domain II predominantly displayed an increased deuterium uptake rate in several key transmembrane helices (TM9, TM13 and TM14), but the degree of the changes in domain II were not as strong as observed in the presence of NADPH.

**Fig. 5:**
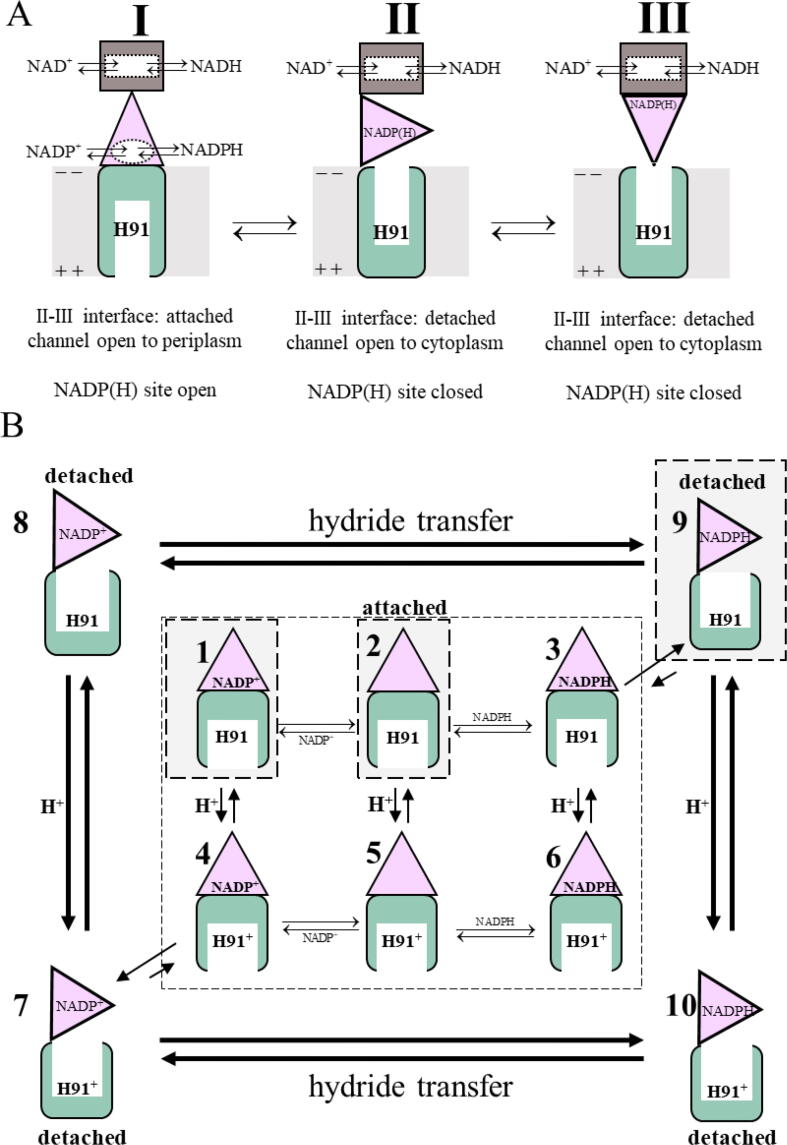
Proposed conformational states of the transhydrogenase described in [6]. Only one protomer is shown. (A). (I) The attached state in which domain III is face-down and the NADP(H) binding site is open for substrate/product exchange; (II) The detached state, in which the NADP(H) binding site is occluded, and the substrate/product is trapped in domain III; (III) The detached state in which domain III is face-up and poised for redox chemistry with NAD(H) in domain I. (B) Equilibria and transitions between the multiple conformational states of the transhydrogenase. The shaded boxes indicate the states proposed to predominate at pH 7.4 for the apo transhydrogenase (species 2), the transhydrogenase bound to NADP^+^ (species 1) and the transhydrogenase bound to NADPH (species 9). Under physiological conditions in vivo, the proton motive force favors His91 protonation from the periplasm, species 4, 5 and 6, and deprotonation to the cytoplasm, species 8 and 9. In the presence of the proton motive force and excess NADP^+^, the catalytic cycle starting with the apo-enzyme corresponds to species 5 → 4 → 7 → 8 → 9 → 3 → 6 → 5.

Next, we analyzed the effects of NAD^+^ binding. Incubation with 1 mM of NAD^+^ resulted in relatively minor conformational changes of the transhydrogenase (Fig. 4C). The major effects were observed in the predicted binding site, the Rossmann fold in domain I, which displayed a decrease in the rate of deuterium uptake. Relatively minor effects were observed in domains II and III.

### 3.5 Effects of enzyme turnover in the presence of NADH plus NADP^+^

In the presence of 1 mM of each NADP^+^ and NADH in the labeling buffer, the enzyme (14.4 µM) will undergo catalytic turnover which should reach equilibrium within a few minutes. After equilibration, the enzyme solution will contain 0.5 mM each NAD^+^, NADH, NADP^+^, and NADPH. We monitored deuterium uptake at equilibrium of the reaction and compared it to the apo-transhydrogenase after 45 min of labeling (Fig. 4D). Domain I displayed a decreased uptake in the region of the NAD(H) binding site, similar to the observed changes in the presence of NAD^+^(Fig. 4C). Domain III, containing the NADP(H) binding site, displayed only minor changes compared to the differences observed in the presence of either NADPH or NADP^+^(Fig. 4A and B). There was a distinct increase in the rate of deuterium uptake in domain II (TM9, TM10, TM13 and TM14), similar to the increased uptake observed in the presence of NADPH.

## 4 Discussion

Here we used HDX-MS to gain further insights into the ligand-induced conformational changes of the *E. coli* membrane-bound transhydrogenase that are central to coupling the redox chemistry to the proton motive force. We investigated the effects of different ligands, NADPH, NADP^+^ and NAD^+^, as well as in-situ generation of NADPH using a combination of NADH and NADP^+^. The most important result is the demonstration of dramatic conformational changes in the transmembrane helices of domain II induced by the binding of NADP^+^ or NADPH to domain III. This shows the allosteric gating of the proton channel by ligand binding of the substrate and product of the reaction. Since membrane-embedded proteins remain a challenging target for HDX-MS, we used a novel cyclic IMS mass spectrometer to improve sequence coverage in membrane segments and analyze minor deuterium uptake differences.

### 4.1 Previous structural studies and proposed conformational changes

The low-resolution (7 Å) X-ray structure of the apo-transhydrogenase from the hyperthermophilic bacterium *Thermus thermophilus* revealed that the two protomers in the dimer are asymmetric [9]. One protomer domain III is “face-up” with the NADP(H) binding site in contact with the NAD(H) binding site in domain I, as required for hydride transfer. However, in the second protomer, domain III is flipped so that the NADP(H) binding site is “face-down” in contact with the cytoplasmic side of the proton channel. It was suggested that domain III must be conformationally dynamic during the catalytic cycle, alternating at least two conformational states.

Considerably more detail was provided by the atomic-resolution cryo-electron microscopy structures of the transhydrogenase from mammalian (ovine) mitochondria in the apo-, NADP^+^-bound, and NADPH-bound states [6]. In this case, the apo-transhydrogenase is symmetric with domain III in the face-down conformation in both protomers (double face-down) as shown in Fig. 1A. Due to the fact, that *E. coli* transhydrogenase too express a linker between dI and dII, it could adapt a double face-down conformation, similar to the mammalian protein [6]. The structure reveals that the domain II-domain III interface is in an “attached” conformation which is proposed to (1) allow rapid exchange of NADPH and NADP^+^ in domain III, and (2) open the proton channel to the mitochondrial intermembrane space (equivalent to the *E. coli* periplasm) while closing the channel gate on the matrix (or bacterial cytoplasm) [6]. This is illustrated in Fig. 5A, I. Addition of either NADPH or NADP^+^, depending on the protonation status of the histidine in the proton channel, is proposed to result in detachment of domain III from domain II (Fig. 5A-II and III) [6]. In the detached conformations, the bound nucleotide is locked in an occluded state within domain III, and it is proposed that the histidine within the proton channel is accessible only to the mitochondrial matrix (equivalent to the bacterial cytoplasm).

The full set of equilibria between ligand-bound species is shown in Fig. 5B. In the structures of the mitochondrial enzyme determined at pH 7.4, the apo-enzyme is expected predominantly occur as state **2** and to a smaller extent as state **5** (as classified in Fig. 5B) [6]. Under physiological conditions, the proton motive force would strongly favor species **5** with the protonated His91^+^. NADP^+^-binding is proposed to result in detachment only if the histidine is protonated (Fig. 5B, **4 → 7**). The structures determined at pH 7.4 show that in the presence of NADP^+^ about 60 % of the population is in the double face-down (attached) configuration, mostly state **1** with some **4** (Fig. 5B) [6]. In about 20 % of the population, domain III is observed only in one protomer (Fig. 5B, **7** and **8**). Presumably, in the second protomer there are multiple detached conformations which are not resolved.

NADPH-binding is proposed to cause detachment only if the histidine is deprotonated (Fig. 5B, **3 → 9**) [6]. In the presence of NADPH at pH 7.4, the heterogeneity of domain III detached conformations is dominant, corresponding mostly to state **9** in Fig. 5B.

### 4.2 HDX-MS shows that NADPH binding induces significant structural changes in *E. coli* transhydrogenase

The highest difference in uptake were observed in the presence of NADPH. NADPH binding at pH 7.4 is expected to favor the detached conformation (Fig. 5B, state **9**) whereas the reference apo-transhydrogenase is in the double face-down/attached conformation, assuming the *E. coli* transhydrogenase is comparable to the mitochondrial enzyme (Fig. 5B, state **2**). The transition from the attached/ face-down conformation to the detached conformation is accompanied by changes in the proton channel to change access to His91 from the periplasm to the cytoplasm. Consistent with this are the pronounced increased deuterium uptake rates in transmembrane helices TM9, TM13 and TM14 which form the core of the proton channel in domain II [1], [6], [35]. Increased deuterium uptake rates can be attributed to changes in structural rigidity or solvent accessibility. The increased deuterium uptake rates are observed along the entire extents of the transmembrane helices across the membrane. This suggests that in the absence of bound ligands in the double face-down/attached conformation the channel may be closed on both ends rather than open to the periplasm as suggested based on the structures of the mitochondrial enzyme [6].

The HDX-MS data may also reflects the binding of NADPH to domain III [1], [4], [6], [16], [36] and the conversion of the binding site from a state that is open to ligand binding to a state in which the bound ligand is occluded. A strong decrease in deuterium uptake is observed at the NADPH binding site, including the KRS motif (PntB: K424, R425, S426), loop D (PntB: P402-L412) and loop E (PntB: K424-F439). In addition, the detached conformation of domain III has a hydrogen bond between the equivalent of residues R350 and Y315 which is not present in the face-down/ attached conformation [6]. The HDX-MS data show a strong decrease in the rate of deuterium uptake in the segments around residues R350 and Y315, consistent with formation of the detached state with occluded NADPH binding site. Moreover, helices hA and hF of domain III displayed a marked decrease in rate of deuterium uptake despite being located on the surface of the domain. In the cryo-EM structures of ovine transhydrogenase, the vast majority of structures showed both domains III of the dimer in the detached form with NADPH bound [6]. The detached state, however, harbors a diverse family of conformations including those in which domain III could rotate by 90°. In this conformation, hA and hF are located close to dI which could result in shielding effects and prevention of deuterium exchange. In the cryo-EM structures of the mitochondrial enzyme, this domain is not resolved due to its flexibility and heterogeneous conformational space [6]. Based on our HDX-MS data, we propose a 90° rotation of domain III in the presence of NADPH, which would explain the observed differential uptake and could represent a transition state during NADPH binding.

In domain I, we detected a diffuse decrease in deuterium uptake, which was less pronounced than in the other domains. This is most likely due to binding of NADPH to the NAD(H) binding site in domain I at the high concentrations used in these studies (1 mM). We observed the same effect in previous studies with these two ligands binding to other proteins [33].

### 4.3 Cyclic IMS HDX-MS analysis of *E. coli* transhydrogenase bound to NADPH

Membrane-embedded protein segments still remain a challenging target to analyze with HDX-MS because of their solvent inaccessibility, hydrophobicity and restricted proteolytic digestion efficiencies [22], [24], [37]. To address these difficulties, we analyzed *E. coli* transhydrogenase together with NADPH on a new cyclic IMS mass spectrometer with enhanced ion optics, detector, and a novel cyclic ion mobility cell. With these improvements, we were able to increase sequence coverage for transmembrane segments from 68% to 73% and could cover segments which were not previously detected. These new segments together with all other peptides displayed significantly higher intensities of peptide cluster peaks. Signal increase was 1 to 1.5 orders of magnitude higher compared to the Synapt G2-Si MS. This increase resulted in better signal-to-noise ratio and led to more precise identification of isotope envelopes of peptide signals.

For the cytosolic domains I and III, we found similar effects compared to the dataset from the Synapt G2-Si MS. The NADP(H) binding site in domain III displayed decreased deuterium uptake rates in essential segments (loop E and loop D) involved in ligand binding [1], [6], [10], [38], [39], whereas there was very little perturbation observed in domain I.

In the Synapt G2-Si MS dataset, there was a marked increase in the deuterium uptake rate in domain II transmembrane helices TM9, TM13 and TM14. In addition, the increased sensitivity revealed changes also in TM4 and TM10 which also form a part of the proton channel and were not present in the Synapt G2-Si data set [1].

### 4.4 Binding of oxidized ligands showed different effects on the protein

Based on the structural data of the mitochondrial transhydrogenase [6] the expectation is that binding of NADP^+^ to domain III should favor the double face-down/attached conformation at pH 7.4 (Fig. 5B, species **1**). However, a substantial fraction (18 %) of structures of the mitochondrial enzyme with bound NADP^+^ show one protomer in which domain III is detached. Hence, our HDX data could reflect these multiple conformational states, if the *E. coli* enzyme behaved similarly.

The HDX-MS data showed NADP^+^ binding resulted in a significant increased rate of deuterium uptake in transmembrane helices TM9 (region near His91), TM13 and TM14 in domain II. The pattern was not identical to that observed in the presence of NADPH. However, the results are more pronounced on the cytoplasmic side of domain II near the interface with domain III. The HDX-MS data could result from a dynamic equilibrium in which the NADP^+^-bound transhydrogenase molecules spend a small fraction of time in a detached conformation. Alternatively, the presence of bound NADP^+^ in domain III, inserted into domain II in the attached conformation, might partially open the channel on the cytoplasmic side. In any event, NADP^+^ binding to domain II does perturb the conformation and increase D_2_O access to portions of domain II.

HDX-MS identified some segments in domain III with increased and others with decreased deuterium uptake rates upon binding NADP^+^. Stretches involved in NADP(H) binding (loop E and loop D) displayed a strong rate decrease due to shielding by the bound NADP^+^. Other segments in this domain (β-sheet D383-G389, helix 4 and the region near R350) exhibited an increased rate of uptake, indicating a more flexible structure with increased solvent access compared to the control condition without ligand. This may be due to perturbation of the interaction between the diphosphate of NADP^+^ with R350 and Y315 resulting in an occluded but more “open” conformation of this portion of domain III. Changes in domain I were relatively small and attributed to minor binding of NADP^+^ to the NAD(H) binding site in domain I.

Next, we analyzed the oxidized form of NAD(H), namely NAD^+^, and its effects on the protein. Binding of this ligand to domain I is not implicated in any perturbation to the proton channel in domain II [1], [6]. The Rossmann fold, the central element of the NAD(H) binding site, exhibited the most pronounced HDX-MS effect correlated with NAD^+^ binding. Domain III displayed only minor changes, likely due to a small degree of cross binding of NAD^+^ to the NADP(H) binding site [33]. No significant changes were observed in domain II. These results support the assumption, that NAD^+^ binding in domain I alone does not affect the activation of the putative proton channel in the transmembrane domain II.

### 4.5 HDX-MS during catalytic turnover

In the presence of 1 mM each of NADH and NADP^+^, the transhydrogenase undergoes catalytic turnover and reaches equilibrium after a few minutes under the experimental conditions. After completion of the reaction, all four ligands are present at a concentration of 0.5 mM and a dynamic equilibrium is established with ligand exchanges and redox chemistry occurring with no net change. We performed this experiment to investigate whether any different conformational states can be detected that are not observed in the presence of individual ligands.

The results obtained under these conditions generally match the data obtained in presence of NADPH (Fig. 4A and D). Domain II displayed a global increase in deuterium incorporation rate including important transmembrane helices TM9, TM10, TM13 and TM14, indicating an active NADPH generation and inducing the putative proton channel to open. In contrast to the NADP^+^ data, we observed increased deuterium uptake across the TM segments and thus full opening of the putative proton channel (TM13, TM14), comparable to the NADPH experiment.

Only minor changes were detected in domain III. During dynamic equilibrium, the NADP(H) binding site binds to and releases NADP^+^/NADPH. This led to reduced differential uptake compared to the NADP^+^ and the NADPH experiment, respectively, as each state is only occupied transiently during the reaction cycle. Our findings could also indicate negative cooperativity between the binding sites, to avoid forming a dead-end complex consisting of either NADPH plus NADH or NADP^+^ plus NAD^+^ [40].

Notably, there was a marked decrease in deuterium uptake in the NAD(H) binding site in domain I, presumably due to shielding by the bound ligand. Segments located at the surface of domain I displayed a minor increase in deuterium uptake. This may be due to active turnover, and only transient binding of NADH and electron transfer to NADPH. Overall, it is clear that the transhydrogenase undergoes major conformational changes during the reaction cycle.

## 5 Conclusion

The current work shows that allosteric gating of the proton channel in domain II induced by specific ligand binding. NADPH binding in domain III clearly induces a full opening of the putative proton channel in domain II. However, the oxidized form NADP^+^ shows different effect on domain II and III. Domain II shows pronounced increase in deuterium uptake on the cytoplasmic side indicating a partial opening of the putative proton channel to this site. Domain III might be in an occluded but more “open” conformation compared to the occluded and closed form with NADPH. These results are in good agreement with the previously proposed function published by Kampjut and Sazanov and give a detailed dynamic insight in the reaction cycle of *E. coli* transhydrogenase [6]. The SELECT SERIES Cyclic IMS mass spectrometer data set allowed an in-depth analysis of the NADPH binding and proves the Synapt G2-Si data in a second biological replicate.

## CRediT authorship contribution statement

**Jonathan Zöller:** Conceptualization, Validation, Formal analysis, Investigation, Data curation, Writing – original draft, Visualization. **Sangjin Hong:** Validation, Formal analysis, Investigation, Data curation, Writing – review & editing. **Martin L. Eisinger:** Conceptualization, Methodology, Writing – review & editing, Supervision. **Malcolm Anderson:** Investigation, Writing – review & editing. **Melanie Radloff:** Investigation, Writing – review & editing. **Kristina Desch:** Formal analysis, Data curation, Writing – review & editing. **Robert Gennis:** Conceptualization, Resources, Writing – review & editing, Visualization, Supervision, Project administration, Funding acquisition. **Julian D. Langer:** Conceptualization, Resources, Writing – review & editing, Visualization, Supervision, Project administration, Funding acquisition.

## Declaration of Competing Interest

The authors declare that they have no known competing financial interests or personal relationships that could have appeared to influence the work reported in this paper.
